# Impact of glioma metabolism-related gene ALPK1 on tumor immune heterogeneity and the regulation of the TGF-β pathway

**DOI:** 10.3389/fimmu.2024.1512491

**Published:** 2025-01-07

**Authors:** YaoFeng Hu, Sen Qin, RuCui Deng

**Affiliations:** ^1^ Department of Neurological Care Unit, The First Affiliated Hospital of YangTze University, Jingzhou, Hubei, China; ^2^ Department of Orthopedics, The First Affiliated Hospital of YangTze University, Jingzhou, Hubei, China

**Keywords:** glioma, metabolic genes, immune microenvironment, ALPK1, prognostic biomarkers

## Abstract

**Background:**

Recent years have seen persistently poor prognoses for glioma patients. Therefore, exploring the molecular subtyping of gliomas, identifying novel prognostic biomarkers, and understanding the characteristics of their immune microenvironments are crucial for improving treatment strategies and patient outcomes.

**Methods:**

We integrated glioma datasets from multiple sources, employing Non-negative Matrix Factorization (NMF) to cluster samples and filter for differentially expressed metabolic genes. Additionally, we utilized Weighted Gene Co-expression Network Analysis (WGCNA) to identify key genes. A predictive model was developed utilizing the optimal consistency index derived from a combination of 101 machine learning techniques, and its effectiveness was confirmed through multiple datasets employing different methodologies. In-depth analyses were conducted on immune cell infiltration and tumor microenvironmental aspects. Single-cell sequencing data were employed for clustering and differential expression analysis of genes associated with glioma. Finally, the immune relevance of the model gene ALPK1 in the context of pan-cancer was explored, including its relationship with immune checkpoints.

**Results:**

The application of NMF, coupled with differential analysis of metabolic-related genes, led to the identification of two clusters exhibiting significant differences in survival, age, and metabolic gene expression among patients. Core genes were identified through WGCNA, and a total of 101 machine learning models were constructed, with LASSO+GBM selected as the optimal model, demonstrating robust validation performance. Comprehensive analyses revealed that high-risk groups exhibited greater expression of specific genes, with ALPK1 showing significant correlations with immune regulation.

**Conclusion:**

This research employed a multi-dataset strategy and various methods to clarify the differences in metabolic traits and immune conditions in glioma patients, while creating an innovative prognostic risk evaluation framework. These results offer fresh perspectives on the intricate biological processes that define gliomas.

## 1 Introduction

Gliomas, malignant tumors originating from neural glial cells, represent one of the most common primary intracranial tumors (1–3). Characterized by high incidence, recurrence rates, mortality, and low curability, they predominantly affect individuals aged 60 to 80 years, with a higher prevalence in males (4). The clinical manifestations of gliomas are diverse and may include headache, nausea, vomiting, and seizures, often leading to cognitive impairment, motor dysfunction, and emotional disturbances, which significantly impact patients’ quality of life and survival (5). Treatment primarily involves surgical resection, complemented by a multimodal approach including radiation therapy and chemotherapy. Despite significant advancements in the diagnosis and treatment of gliomas in recent years, patient prognoses remain poor, particularly for those with high-grade gliomas, where the median survival time is still relatively short (6, 7). Therefore, exploring the molecular subtyping of gliomas, identifying new prognostic biomarkers, and understanding the characteristics of their immune microenvironment are crucial for improving treatment strategies and patient outcomes.

Nucleotide metabolism plays a vital role in cellular survival and proliferation, significantly impacting tumor development and progression (8). Abnormalities in nucleotide metabolism can promote rapid tumor cell proliferation and may affect the sensitivity of these cells to treatment (9). Previous research has indicated that alterations in nucleotide metabolism are strongly associated with the progression of glioma, especially in the realms of cell cycle control, DNA repair mechanisms, and energy metabolism (10). However, the specific impact of nucleotide metabolism on the prognosis of glioma patients and its potential as a novel therapeutic target remain to be fully explored.

This study aims to integrate multi-source RNA sequencing data and utilize various bioinformatics approaches to reveal differences in metabolic characteristics and immune status among glioma patients, thereby constructing a reliable prognostic risk scoring system. We employed Non-negative Matrix Factorization (NMF) technology to cluster the merged glioma Bulk RNA-seq dataset based on nucleotide metabolism gene expression patterns, identifying two optimal clusters (11). These two clusters exhibited significant differences in survival rates and age distributions. Following Weighted Gene Co-expression Network Analysis (WGCNA) analysis, six modules associated with clinical pathological features were identified (12). By constructing and evaluating 101 machine learning prognostic models, we ultimately selected the LASSO+GBM combination model, which demonstrated good performance in both training and validation sets, as well as validation in an independent dataset.

By employing this model, we computed risk scores for patients and identified connections with genes related to apoptosis and the cell cycle, indicating potential dysregulation in the mechanisms that regulate tumor proliferation. Further investigations revealed significant differences in immune cell infiltration levels across different clusters and risk categories, providing valuable insights into the mechanisms of glioma immune evasion and the exploration of immunotherapeutic strategies. In light of the absence of single-cell data specifically on glioma immune checkpoint inhibitors, we analyzed single-cell data from lung cancer to infer the distribution and functional characteristics of immune cells, thereby recognizing various cell subpopulations and their functional distinctions. A comprehensive analysis across multiple cancers revealed a notable relationship between the ALPK1 gene and different immune checkpoints, alongside its link to the prognosis of patients with glioma. Elevated levels of ALPK1 expression were associated with heightened infiltration of immune cells in several cancer types, indicating its potential significance in modulating the immune microenvironment of gliomas and positioning it as a promising target for forthcoming immunotherapeutic strategies.

In summary, this research examined gene expression data related to gliomas using various approaches, identified variations in metabolic features and immune conditions, and developed a dependable prognostic risk scoring system. This work offers a fresh viewpoint for comprehending the biological mechanisms of glioma and devising targeted medical strategies, thus aiding in the enhancement of patient survival rates.

## 2 Material and methods

### 2.1 Data acquisition and preprocessing

We employed the “TCGAbiolinks” R package to acquire bulk RNA-sequencing (RNA-seq) data from 704 glioma specimens along with 5 adjacent normal specimens from The Cancer Genome Atlas (TCGA) (13). Furthermore, we accessed additional bulk RNA-seq data comprising 693 glioma samples (CGGA_693) and 325 glioma specimens (CGGA_325) from the Chinese Glioma Genome Atlas (CGGA, http://www.cgga.org.cn/). The single-cell sequencing datasets were obtained from the Tumor Immune System Interaction and Cancer Heterogeneity Database 2 (TISCH2, http://tisch.comp-genomics.org/home/), focusing on three glioma datasets (GSE103224, GSE131928, GSE138794). Additionally, we downloaded a single-cell dataset related to lung cancer (post-immunotherapy), GSE207422, from the Genomics Expression Omnibus (GEO, https://www.ncbi.nlm.nih.gov/geo) (14). We merged the glioma bulk RNA-seq data utilizing the Combat function from the “sva” R package. All datasets were meticulously reviewed to eliminate any incomplete or NA values that might interfere with the analysis outcomes. In instances where the data distribution range was excessively wide, log2 transformation was applied. The analysis of all bulk RNA-seq data was conducted in the format of Transcripts Per Kilobase of exon model per Million mapped reads (TPM). The datasets used in this study are publicly available and do not require ethical review. The nucleotide metabolism-related genes, glycolysis-related genes, amino acid metabolism-related genes, and lipid metabolism-related genes analyzed in this paper were downloaded from the “msigdbr” R package.

### 2.2 Non-negative matrix factorization

Using nucleotide metabolism genes, we first performed NMF clustering on the merged glioma bulk RNA-seq dataset. The NMF technique, employing the ‘brunet’ method, was applied to classify the samples. The number of clusters (k) was varied from 2 to 10 to identify the optimal fit. The advantage of NMF is its ability to reduce the dimensionality of high-dimensional data through non-negative constraints while preserving the natural clustering structure of the data, which is suitable for extracting meaningful features from complex biological data. We evaluated the most suitable cluster number (k) through a collaborative assessment of cophenetic correlation, residuals, dispersion, residual sum of squares (RSS), explained variance (evar), silhouette score, and sparsity. The specific criteria for selection included maximizing the cophenetic correlation to enhance the consistency between clustering results and the original data; optimizing dispersion and silhouette scores to improve clustering distinguishability and quality; selecting the last k value with a significant reduction in residuals and RSS to ensure model fit; and focusing on a notable increase in explained variance while seeking a balance point for sparsity to ensure the interpretability of the clustering results. Subsequently, we analyzed the differences in overall survival (OS) and age among the identified clusters.

### 2.3 Comparison of metabolic genes and functional pathways among clusters

We examined the differences in expression of genes associated with glycolysis, amino acid metabolism, and lipid metabolism across various clusters, visualizing our findings through heatmaps. Next, we utilized the “limma” package to carry out a differential expression analysis of genes between the clusters and performed Gene Set Enrichment Analysis (GSEA) based on the differentially expressed genes (DEGs), illustrating both the upregulated and downregulated pathways (15, 16). The analysis of data was supported by the “clusterProfiler” package, while the visual representations were created using the “enrichplot” package (17, 18). Information regarding pathway-related terms was obtained from the Kyoto Encyclopedia of Genes and Genomes (KEGG) (19).

### 2.4 Weighted gene co-expression network analysis

We executed WGCNA using the “WGCNA” R package (20). The advantage of WGCNA is the ability to construct gene co-expression networks, identify functionally relevant gene sets through modular analysis, and correlate them with clinical features, thereby discovering genes or gene modules that are closely related to disease prognosis. Initially, we removed genes with low expression levels or minimal variability across all samples. Next, we constructed a correlation matrix and an adjacency matrix, determining the power parameter β based on scale independence and mean connectivity. We then generated a Topological Overlap Matrix and constructed the co-expression network. Module partitioning and merging of similar modules were performed using the Dynamic Tree Cut method. Following this, we analyzed the correlation of each module with variables such as Age, Alive, Dead, OS time, and Cluster, and visualized these relationships. Core genes were further chosen from the module that was most closely associated with the clusters, adhering to the criteria of Module Membership (MM) > 0.6 and Gene Significance (GS) > 0.4. Moreover, we performed enrichment analysis on the genes found within each module, with the exception of the gray module, utilizing the “clusterProfiler” version 4.0 R package to visualize the outcomes.

### 2.5 Construction of 101 machine learning prognostic models

We utilized an integrated dataset as the training set, with GSE102073 and GSE26712 serving as validation sets. Employing the “Mime” R package, we executed a combinatorial analysis of 10 machine learning algorithms, resulting in a total of 101 distinct prognostic modeling combinations (21). The gene list input for the calculations comprised core genes identified through WGCNA. Using the integrated functionalities of the “Mime” R package, we calculated the consistency index (C-index) for each algorithm across different datasets, along with the Mean C-index across all cohorts and the Mean C-index in the validation cohorts. We selected the algorithm combination corresponding to the maximum Mean C-index in the validation cohorts as the final prognostic model algorithm. Following this, we calculated risk scores for all patients by utilizing the algorithm and categorized each dataset into high-risk and low-risk tiers according to the median score values. The differences in prognostic levels between the two risk categories within each dataset were evaluated through the Kaplan-Meier method. We validated the performance of the algorithms across different datasets by employing receiver operating characteristic (ROC) curves at 1, 3, and 5 years. Furthermore, we conducted a meta-analysis across three datasets.

### 2.6 Exploration of risk scores, clinical features, and carcinogenic pathways

Initially, we utilized a Sankey diagram to display the distribution patterns of samples across varied clusters and survival statuses within the two risk categories. Subsequently, we conducted an analysis of differential gene expression between the two risk categories and assessed the variations in activity within classical cancer-related pathways. We employed heatmaps to visualize the differences in both pathway activities and gene expression across the two risk categories. We also presented correlation heatmaps to illustrate the relationships between risk scores and apoptosis-related genes, as well as cell cycle-related genes. Additionally, we analyzed the top ten Single Nucleotide Polymorphism (SNP) genes ranked within each risk group. Finally, we demonstrated the correlation between risk scores and various classical tumor pathways.

### 2.7 Immune-related analyses

We commenced our analysis by examining the differences in infiltration levels of 22 immune cell types across various clusters and between risk groups. We utilized the “IOBR” R package (https://github.com/IOBR/IOBR) to perform immune cell infiltration analyses of bulk RNA-seq datasets through built-in algorithms such as CIBERSORT, EPIC, MCP-Counter, quanTIseq, TIMER, and xCell. Furthermore, we assessed differences in tumor microenvironment scores between the different risk groups using the ESTIMATE algorithm. Ultimately, we acquired Immunophenoscore (IPS) data for every sample from The Cancer Immunome Atlas (TCIA) (https://tcia.at/home) and examined the differences in IPS scores that could predict the effectiveness of PD-L1 or CTLA-4 inhibitor therapies among various risk groups.

### 2.8 Single-cell sequencing data analysis

The preprocessing of single-cell sequencing data was accomplished utilizing the Seurat pipeline. Given that the TISCH2 database had previously undergone basic quality control checks, it was deemed unnecessary to implement further quality control measures. The “Harmony” R package was employed to mitigate batch effects for the integration of multiple samples. Annotation was conducted using information sourced from the TISCH database. To evaluate the model genes, we utilized the AddModuleScore function within Seurat, allowing us to examine the distribution of these scores across various cell populations. Moreover, we isolated malignant cells for additional dimensionality reduction and clustering, which was subsequently followed by differential and enrichment analyses for each subpopulation. The Uniform Manifold Approximation and Projection (UMAP) algorithm was applied for this dimensionality reduction and clustering process. In our focus on the non-small cell lung cancer dataset, GSE207422, we assessed the distribution of cases that were treatment-naïve (NE), treatment-responsive (MPR), and treatment-resistant (NMPR) in accordance with the provided treatment data. We analyzed the disparities in model gene scores across the distinct treatment groups, allowing us to categorize cells into high and low scoring groups. GSEA was then implemented to evaluate differences in pathways between these two groups. For three additional glioma datasets, we conducted integration and applied similar analytical methods.

### 2.9 Pan-cancer and immune analysis based on the model gene ALPK1

We first conducted an analysis of the association between ALPK1 and various immune modulators—including receptors, MHC molecules, immune stimulators, and chemokines—across 33 different tumor types. Subsequently, we focused on visualizing the Pearson correlation coefficients between ALPK1 and four immune checkpoints—CD274 (PD-L1), CTLA-4, LAG-3, and PDCD1 (PD-1)—in glioblastoma (GBM) and lower-grade glioma (LGG). We utilized the single-sample Gene Set Enrichment Analysis (ssGSEA) method to investigate the association between ALPK1 expression and the infiltration levels of 28 immune cell types associated with tumors across 33 different types of tumors. The correlation coefficients were derived using Pearson correlation methods, and statistical significance was calculated with p-values.

The glioma dataset was divided into categories reflecting high and low expression levels according to the median expression of ALPK1. First, we investigated the differences in immune modulators between these two categories and illustrated the results using heatmaps. Following that, we collected relevant information about the anti-cancer immunity cycle from the literature and evaluated the differences in the various stages of this cycle between the two categories (22). Following this analysis, we evaluated the expression differences of immune cell-associated effectors across the groups. Ultimately, a correlation analysis was conducted between ALPK1 and molecules that suppress immune responses.

### 2.10 Statistical analysis

All statistical evaluations were performed utilizing R software (version 4.1.3). The “clusterProfiler” package facilitated the data analysis, while visual representations were produced with the help of the “enrichplot” package. The “limma” package was employed for the analysis of differential gene expression among clusters. Unless stated differently, all figures were generated using “ggplot2.” A p-value threshold of < 0.05 was regarded as statistically significant (* p < 0.05; ** p < 0.01; *** p < 0.001; **** p < 0.0001).

### 2.11 Cell culture

The following cell lines were utilized in this study for *in vitro* experiments: Neuroependymal Hypothalamic Astrocytoma (NHA) cells, HS683, LN229, U87MG, and U251MG, sourced from the Cell Bank of the Chinese Academy of Sciences in China. NHA served as the normal control cell line, whereas the other lines were classified as tumor cell lines. Culturing was performed in Dulbecco’s Modified Eagle Medium (DMEM; Hyclone, USA) for NHA, HS683, LN229, and U251MG, while U87MG was maintained in Minimum Essential Medium (MEM; Hyclone, USA). Each culture medium was enriched with 10% fetal bovine serum (FBS; Hyclone, USA) and 1% penicillin-streptomycin solution (Keygen, China) to prevent bacterial proliferation. The cells were kept in a humid atmosphere at 37°C with 5% CO₂ to promote logarithmic growth.

### 2.12 Transfection

Transfection experiments were conducted on the HS683 and LN229 cell lines using siRNA (Sangon, China) for the transient knockdown of the ALPK1 gene, with a negative control (NC) serving as the control group. The cells were first placed in six-well plates and permitted to achieve about 80% confluence. A suitable amount of Opti-MEM reduced serum medium (Thermo, USA) was used to dissolve Lipofectamine 3000 (Thermo, USA) and siRNA, which was allowed to sit for 5 minutes. Following this, the two components were mixed and incubated for 20 minutes before adding the mixture to the six-well plates. The medium was replaced 5 hours post-transfection.

### 2.13 Total RNA extraction and RT-qPCR

Cells from each group were digested with trypsin and collected in centrifuge tubes. After centrifugation and washing, the resulting pellets were obtained. To lyse cell structures and inhibit RNase activity, 950 μL of Trizol (Takara, Japan) was added. The mixture was allowed to stand for 5 minutes, followed by the addition of 150 μL of chloroform (Sinopharm, China), which was thoroughly mixed by vortexing. The mixture was then centrifuged at 12,000 g for 5 minutes at 4°C, and the supernatant was carefully collected. An equal volume of isopropanol (Sinopharm, China) was added to promote RNA precipitation. After another 5-minute centrifugation at 12,000 g and 4°C, the precipitate was washed with 1 mL of 75% ethanol or anhydrous ethanol and allowed to dry naturally. All operations were performed under RNase-free conditions, and the concentration of the extracted RNA, as well as DNA and protein contamination, were assessed.

Genomic DNA was removed using the PrimeScript RT Master Mix (TaKaRa, Japan), and appropriate reaction mixtures were prepared according to the manufacturer’s instructions and the measured RNA concentration. Complementary DNA (cDNA) was synthesized through reverse transcription. Real-time quantitative PCR (qPCR) analysis was conducted on a Roche480 PCR system (Roche, Switzerland) using SYBR GreenER Supermix (TaKaRa, Japan), following the manufacturer’s protocol. All samples and reagents were pre-mixed before analysis. Each experimental group included three technical replicates, with β-actin serving as the internal control gene.

### 2.14 Cell counting kit-8 assay

Twenty-four hours post-transfection, cells were seeded into 96-well plates at a density of 4,000 cells per well and allowed to adhere, with three technical replicates for each group. The CCK8 reagent (KeyGEN, China) was prepared according to the manufacturer’s instructions, mixed with the culture medium, and adjusted to a final volume of 100 μL per well. The plates were then shielded from light and placed in a cell culture incubator. After 2.5 hours, the absorbance at 450 nm was measured using a spectrophotometer, with measurements repeated at various time points.

## 3 Results

### 3.1 Differential analysis of NMF and clustering

**Figure 1:**
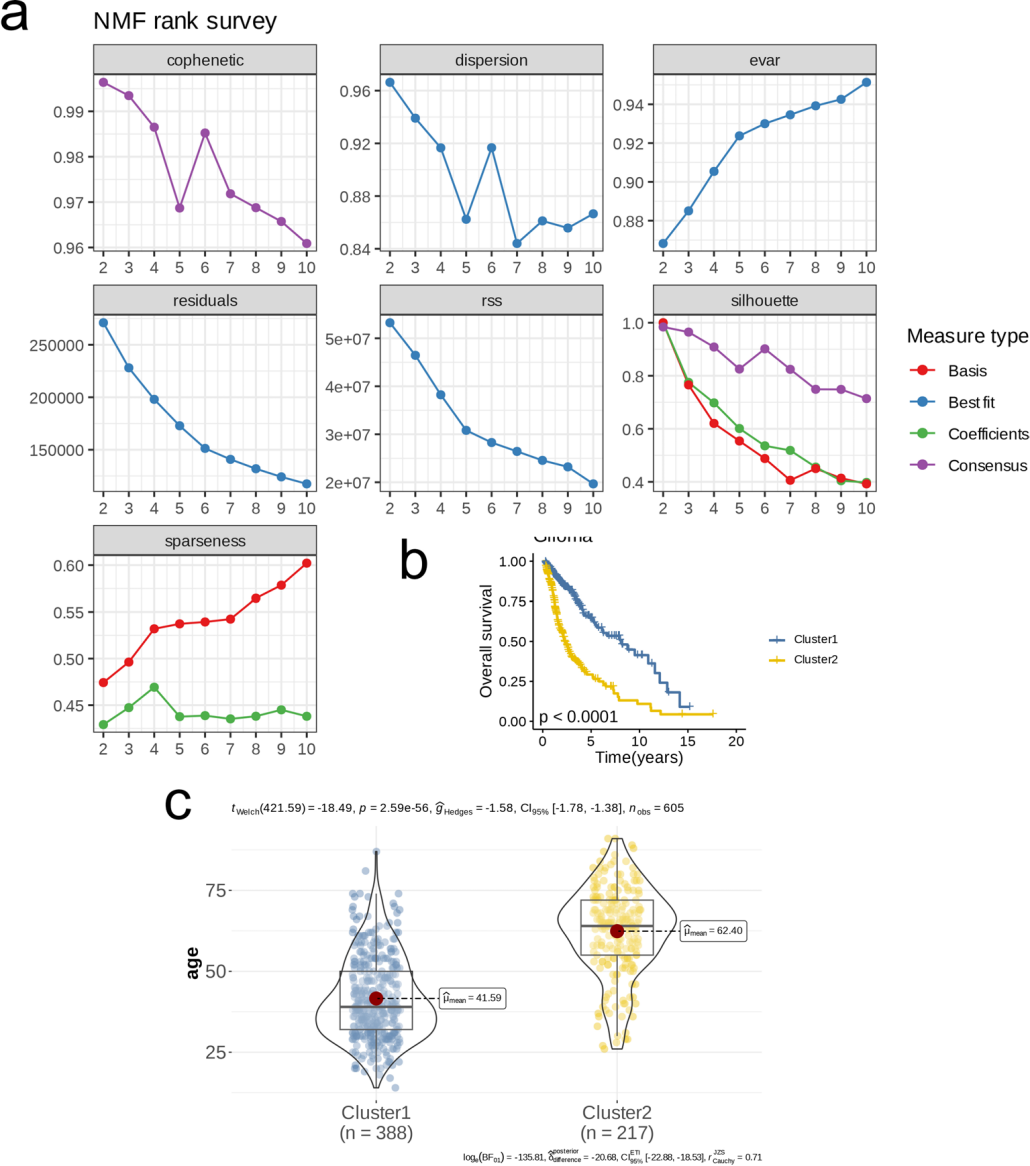
Nucleotide metabolism subclusters and prognosis in TCGA- LGG/GBM. **(A)** Cophenetic distributions, residual sum of squares (RSS), and dispersion indices for ranks 2–10. **(B)** Overall Kaplan-Meier survival curves for both subclusters. **(C)** The age distribution between two subclusters.

**Figure 2:**
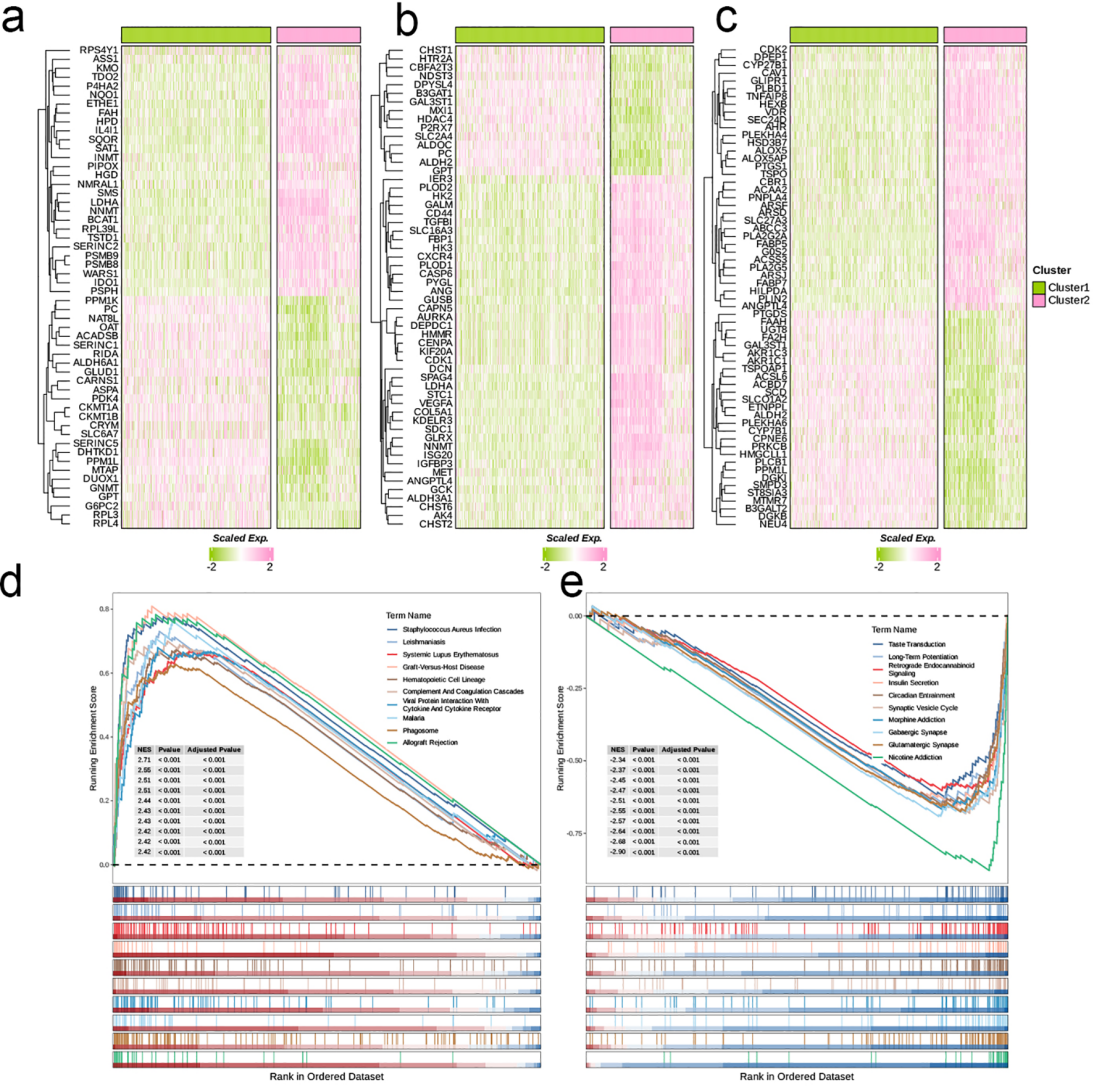
Crosstalk between nucleotide metabolism subclusters and key metabolic pathways. **(A)** Differences in glycolysis-related genes between subclusters. **(B)** Differences in amino acid metabolism-related genes between subclusters. **(C)** Differences in lipid metabolism-related genes between subclusters. **(D)** Gene set enrichment analysis (GSEA) reveals pathways downregulated in subtype C2 relative to C1. **(E)** GSEA reveals pathways upregulated in subtype C2 relative to C1.

We determined the optimal number of clusters (k) to be 2 through multiple metrics, categorizing all patient samples into two distinct classes. At this point, all metrics demonstrated ideal outcomes (**Figure 1A**). Subsequently, we conducted OS analysis for the two clusters, revealing that patients in cluster 2 had a significantly lower survival rate compared to those in cluster 1 (p < 0.0001, **Figure 1B**). Furthermore, there was a notable difference in age distribution between the two clusters, with an average age of 41.59 years in cluster 1 and 62.40 years in cluster 2 (p < 0.001, **Figure 1C**). We then analyzed the expression differences of glycolysis-related genes, amino acid metabolism-related genes, and lipid metabolism-related genes between the clusters, visualizing the results with heatmaps. Notably, cluster 2 exhibited a higher expression of glycolysis-related genes, amino acid metabolism-related genes, and lipid metabolism-related genes compared to cluster 1 (**Figures 2A–C**). GSEA of DEGs between the two clusters indicated that pathways such as the Complement and Coagulation Cascades (NES = 2.43, p < 0.001) and Viral Protein Interaction with Cytokine and Cytokine Receptor (NES = 2.43, p < 0.001) were upregulated in cluster 1 (**Figure 2D**), while pathways including Insulin Secretion were downregulated in cluster 1 (NES = -2.47, p < 0.001, **Figure 2E**).

### 3.2 Core gene selection

**Figure 3:**
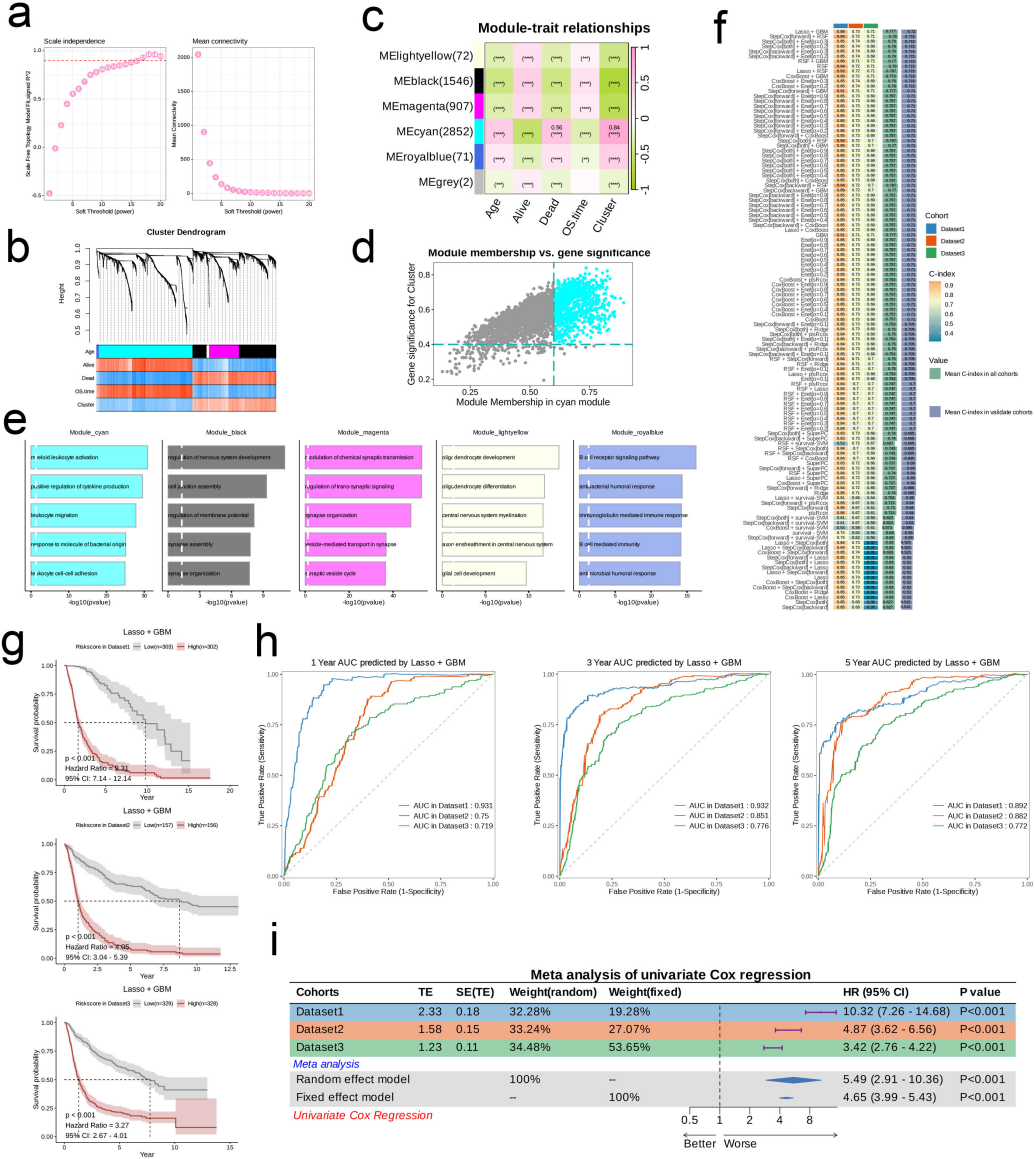
Models Construction based on nucleotide metabolism subclusters. **(A)** Analysis of network topology for different soft-threshold power. The left panel shows the impact of soft-threshold power (power = 16) on the scale-free topology fit index; the right panel displays the impact of soft-threshold power on the mean connectivity. **(B)** Cluster dendrogram of the co-expression modules. Each color indicates a co-expression module. **(C)** Module-trait heatmap displaying the correlation between module eigengenes and clinical traits. **(D)** Correlation between module membership and gene significance in the turquoise module. Dots in color were regarded as the hub genes of the corresponding module (MM > 0.6 & GS > 0.4). **(E)** Top five enriched GO terms of module genes in each module except for the grey. **(F)** A total of 101 kinds of prediction models fitted in TCGA- LGG/GBM (Dataset1) and verified in the other two validation cohorts (GSE102073 [Dataset2] and GSE26712 [Dataset3]). The model was ordered by the average of the C-index of all datasets. The optimal model developed by “StepCox[forward]+GBM” was utilized in subsequent analyses. **(G)** Survival differences between two groups in the three datasets. **(H)** Time-dependent ROC analysis of the model in the three datasets. **(I)** Meta analysis of univariate Cox regression across the three datasets.

We employed WGCNA for preliminary gene selection in subsequent machine learning applications. To ensure scale-free network characteristics, we set the threshold for scale independence at 0.9, resulting in a soft threshold (β) of 16. The average connectivity assessment indicated that the sparsity of the network was appropriate under this soft threshold (**Figure 3A**). We generated a clustered dendrogram of co-expression modules to illustrate the clustering hierarchy and effectiveness (**Figure 3B**). In total, six modules were identified, with almost every module showing a highly significant correlation with clinical pathological features. The modules MElightyellow, MEblack, and MEmagenta displayed similar correlation trends with clinical pathological features, while MEcyan and MEroyalblue showed similar trends in correlation. Notably, MEcyan was significantly positively correlated with Cluster (R = 0.84, p < 0.00001) and also exhibited a significant positive correlation with the Dead parameter (R = 0.56, p < 0.00001, **Figure 3C**). Further analysis and visualization of the MEcyan genes were performed (**Figure 3D**). The analysis of enrichment for each module indicated that Module_cyan was mainly associated with pathways related to the immune system, including the activation of myeloid leukocytes, the enhancement of cytokine production, and the movement of leukocytes. Module_black was primarily enriched in pathways that relate to the assembly of cell junctions, while Module_magenta was associated with pathways that play a role in the modulation of chemical synaptic transmission and the organization of synapses. In addition, Module_lightyellow focused on pathways pertinent to the development of oligodendrocytes and the myelination of the central nervous system, whereas Module_royalblue was connected to pathways linked to B cell receptor signaling and immune responses mediated by immunoglobulins (**Figure 3E**).

### 3.3 Construction of 101 machine learning prognostic models

Utilizing the training dataset, we conducted a total of 101 prognostic models by employing machine learning methods and selecting among them, subsequently ranking the models according to the mean C-index calculated across all datasets. The comparison revealed that the combination of LASSO and Gradient Boosting Machine (GBM) algorithms achieved the highest mean C-index in validation cohorts (0.72). Under this algorithm, the training set C-index was 0.89, validation set 1 C-index was 0.73, and validation set 2 C-index was 0.72, indicating that this model combination possesses good accuracy and generalizability, effectively mitigating the risk of overfitting (**Figure 3F**). The risk score for every patient was determined through this algorithm, and the datasets were categorized into high-risk and low-risk groups according to the median score of each dataset. In all three datasets, the survival rates for the high-risk group were notably lower compared to those of the low-risk group, suggesting that the risk score serves as a negative prognostic indicator (HR > 3, p < 0.001, **Figure 3G**). Furthermore, the ROC curves at 1, 3, and 5 years for the three datasets indicated that this combination of models exhibits strong diagnostic performance (AUC > 0.7, **Figure 3H**). Results from the meta-analysis revealed considerable heterogeneity across the three datasets; in these scenarios, the risk associated with the high-risk group was markedly elevated compared to the low-risk group in each dataset (HR > 1, p < 0.001), illustrating the robustness and generalizability of our model (**Figure 3I**).

### 3.4 Exploration of risk scores, clinical features, and carcinogenic pathways

**Figure 4:**
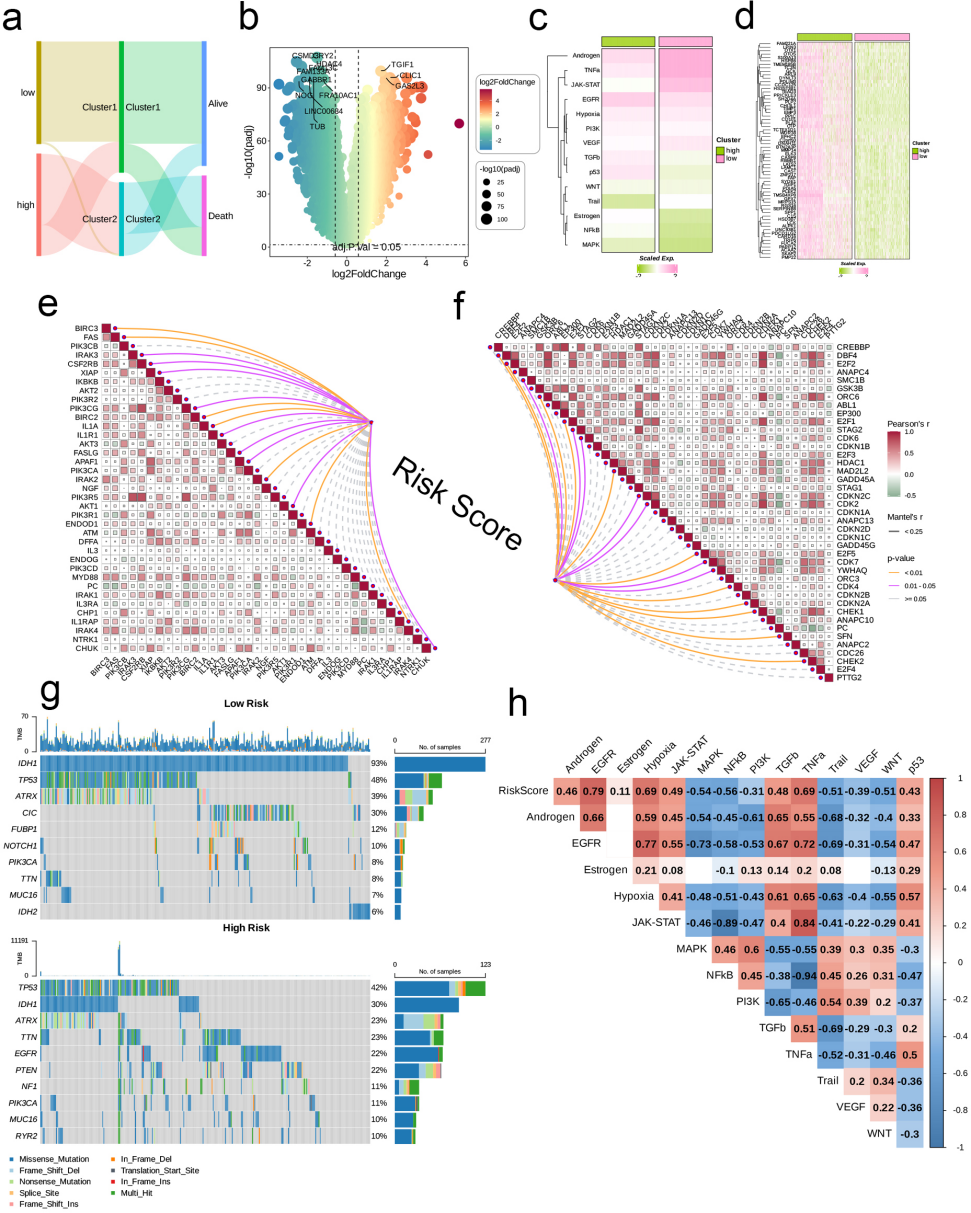
Associations between risk scores, clinical features, and oncogenic pathways in TCGA- LGG/GBM. **(A)** Distribution of risk groups among nucleotide metabolism subclusters and survival samples. **(B)** Differential genes between risk groups. **(C)** Activity differences in classic cancer-related pathways between risk groups. **(D)** Relationships between risk groups and gene expression levels. **(E)** Correlation of risk scores with apoptosis-related genes. **(F)** Correlation of risk scores with cell proliferation-related genes. **(G)** Distribution of the top 10 genes with the highest mutation frequencies across different risk groups. **(H)** Correlation of risk scores with enrichment scores of different classic tumor pathways.

Initially, we employed a Sankey diagram to represent the trends in sample distribution among various clusters and survival statuses within the two identified risk groups. Cluster 1 primarily encompassed patients from the low-risk category, while cluster 2 was predominantly populated by patients from the high-risk group, who exhibited a greater mortality rate compared to those in cluster 1. The survival trends observed across different clusters aligned with those seen between the risk groups (**Figure 4A**). Notably, genes that were expressed at elevated levels in the high-risk group relative to the low-risk group were documented (**Figure 4B**). In general, pathways including Androgen, TNFa, JAK-STAT, EGFR, Hypoxia, PI3K, and VEGF displayed significant activity across both risk categories; nevertheless, the intensity of activation of these pathways differed. The low-risk group manifested considerably higher activity in the Androgen, TNFa, JAK-STAT, VEGF, and Trail pathways, while the high-risk group showed increased activity in other pathways (**Figure 4C**). Among the 63 genes selected for modeling, nearly all were markedly overexpressed in the high-risk cohort (**Figure 4D**). Moreover, we investigated the relationship between risk scores and genes related to apoptosis as well as those associated with the cell cycle. Analysis revealed a significant relationship between risk scores and genes like BIRC3, FAS, BIRC2, IL1A, IRAK2, ENDOD1, and IL1RAP (p < 0.01), in addition to a significant connection with IRAK3, CSF2RB, and XIAP (p < 0.05), which are implicated in apoptosis. Furthermore, risk scores exhibited significant associations with cell cycle-related genes such as DBF4, E2F2, and SMC1B (p < 0.05, **Figures 4E, F**). Analysis of mutations indicated that the IDH1 mutation was the most common in the low-risk cohort, appearing in 93% of cases, whereas TP53 mutations were the most frequent in the high-risk group at 42%. Both risk groups exhibited mutations in TP53, IDH1, TTN, ATRX, and PIK3CA. Among the various types of mutations, missense mutations emerged as the most frequent, followed by multi-hit mutations, while other mutation forms were relatively rare (**Figure 4G**). Additionally, we established a connection between risk scores and several established tumor pathways. The most substantial positive association was found between risk scores and EGFR (R = 0.79), while the strongest negative association was noted with NFkB (R = -0.56, see **Figure 4H**).

### 3.5 Immune-related analysis

**Figure 5:**
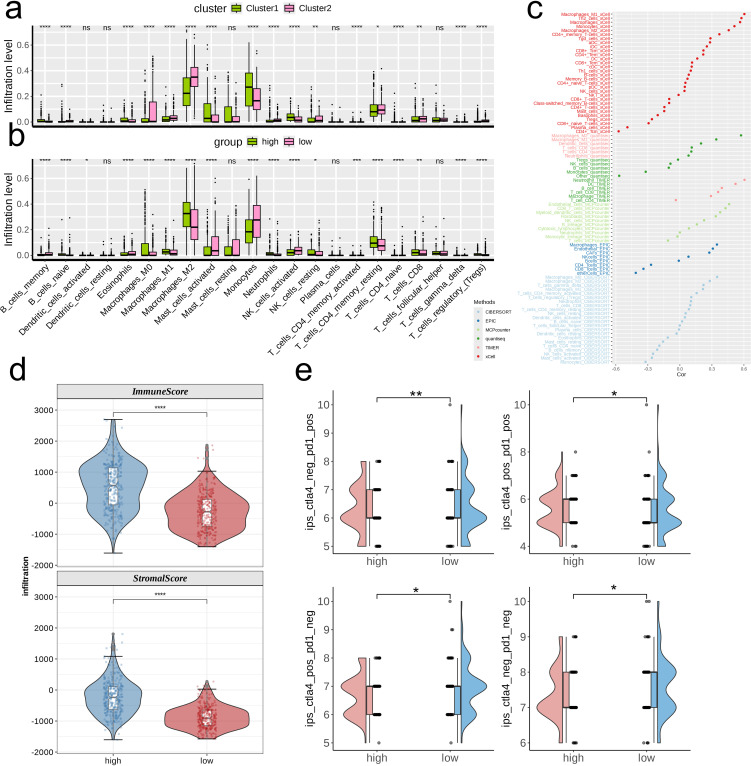
Investigation of the correlation between risk score and the immune microenvironment of TCGA- LGG/GBM. **(A, B)** Differences in infiltration levels of 22 immune cell types between nucleotide metabolism subclusters and between risk groups. **(C)** Correlation of risk scores with various immune cells as revealed by seven different algorithms. **(D)** Differences in tumor microenvironment scores between different risk groups as revealed by the ESTIMATE algorithm. **(E)** Differences in IPS scores predicting effectiveness of PD-L1 or CTLA-4 inhibitor treatments between different risk groups. IPS score of each TCGA- LGG/GBM sample was acquired from the TCIA (https://tcia.at/home). ns, not significant; *p < 0.05; **p < 0.01; ***p < 0.001; ****p < 0.0001.

We first analyzed the differences in infiltration levels of 22 immune cell types across various metabolic clusters and risk groups. In general, the patterns observed in infiltration levels showed distinct variations among clusters and risk categories, with the majority of immune cell types demonstrating significant differences across the various clusters and risk categories. Specifically, cluster 2 exhibited a broader range of immune cell types with heightened infiltration when compared to cluster 1, while the high-risk category revealed a greater quantity of immune cell types with increased infiltration in contrast to the low-risk category (**Figures 5A, B**). We employed six varied algorithms to clarify the relationship between risk scores and different immune cells (**Figure 5C**). The findings from the ESTIMATE analysis revealed that both the Immune score and the Stromal score were considerably elevated in the high-risk category compared to the low-risk category (p < 0.0001, **Figure 5D**), indicating a more abundant infiltration of immune and stromal cells within the tumor microenvironment of patients categorized as high-risk. Additionally, individuals in the high-risk category were more likely to gain advantages from immunotherapy based on differing levels of PD-L1 or CTLA-4 expression (p < 0.05, **Figure 5E**).

### 3.6 Single-cell sequencing data analysis

**Figure 6:**
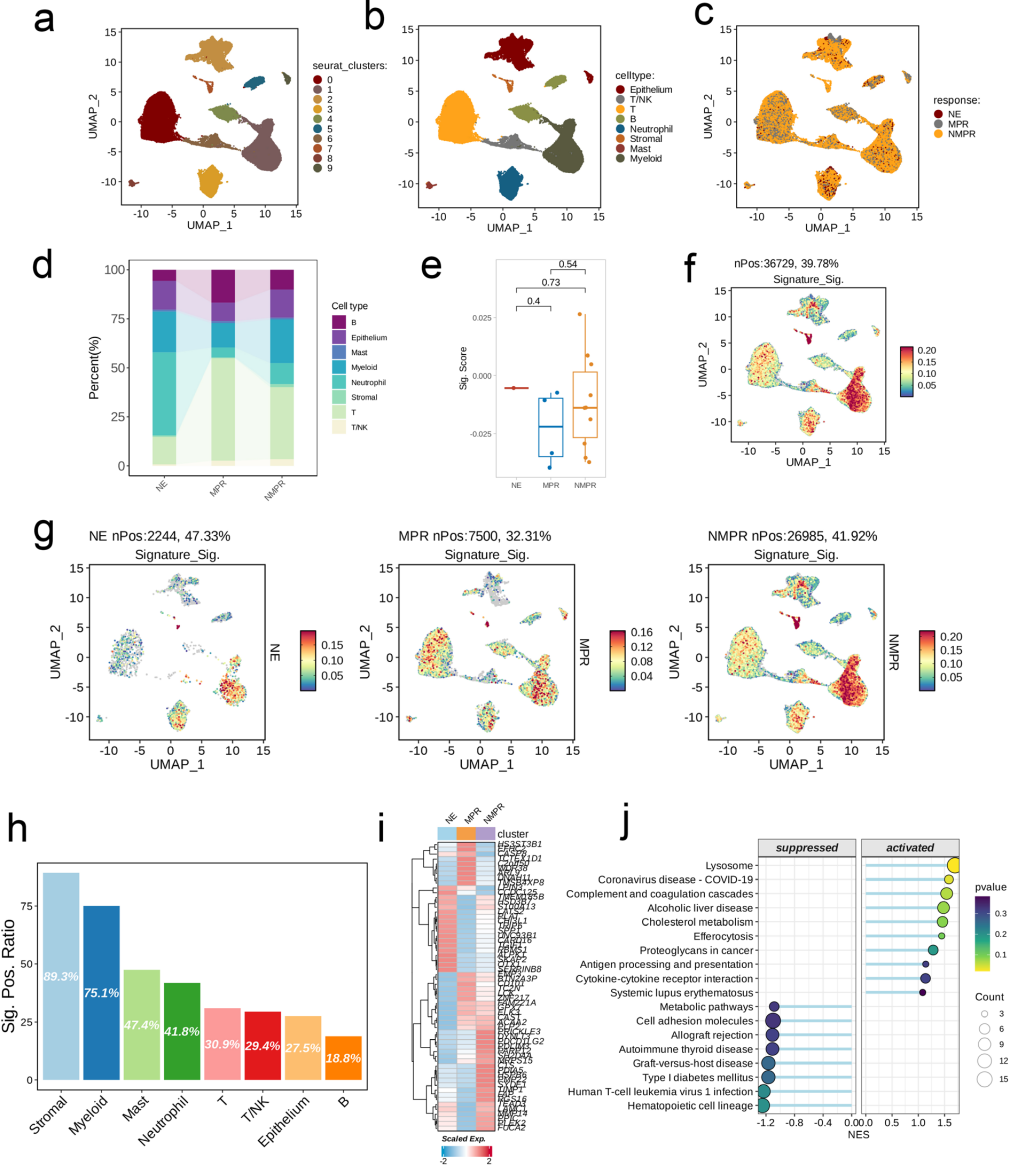
Single-cell analysis of risk score in immunochemotherapy treated scRNA-seq cohort. **(A–C)** UMAP visualization of 92,330 cells from the public NSCLC scRNA-seq cohort treated with immunochemotherapy. A total of ten subpopulations were identified under the resolution of 0.6 and manually annotated to eight meta-clusters based on the cranial markers provided in the original publication. **(D)** Differences in the abundance of cell types across different groups. **(E)** Distribution of the signature scores between groups. The signature score was calculated by the (*AddModuleScore)* function implemented in the “Seurat” package based on the genes derived from the model from the machine-learning pipeline. **(F, G)** UMAP visualization of the signature scores across cell types **(F)** and different groups **(G)**. **(H)** The positive ratio of the signature across each cell type. **(I)** The differences in the abundance of signature genes across different groups in all patients. **(J)** GSEA reveals significantly altered pathways in cells with high signature scores compared to those with low scores.

Due to the absence of single-cell datasets for gliomas treated with immune checkpoint inhibitors, we leveraged datasets related to lung cancer for our analysis. Using the built-in dimensionality reduction clustering algorithm in Seurat, we classified all 92,330 cells into 10 clusters at a resolution of 0.6 (**Figure 6A**). Subsequently, we annotated the cells into eight categories based on biological marker genes: Epithelium, T/NK, T, B, Neutrophil, Stromal, Mast, and Myeloid (**Figure 6B**). The NMPR cell category represented the majority of the cell population, while NE and MPR cells were distributed sparsely (**Figure 6C**). Within the NE group, Neutrophils accounted for the largest proportion; conversely, T cells comprised the highest proportion in the MPR and NMPR groups (**Figure 6D**). We then presented the overall signature scores for the NE, NMPR, and MPR groups (**Figure 6E**). Notably, high signature scores were predominantly found in the Myeloid cell subpopulation (**Figure 6F**). Specifically, within the NE group, high signature scores were primarily concentrated in the Myeloid and Neutrophil subpopulations; in the MPR group, they were found mainly in the Myeloid and T cell subpopulations; and in the NMPR group, high signature scores were primarily associated with Myeloid and Stromal cell subpopulations (**Figure 6G**). The proportion of signature-positive cells varied across different subpopulations, with the Stromal cell subpopulation exhibiting the highest proportion (89.3%), while the B cell subpopulation had the lowest (18.8%, **Figure 6H**). The heatmap illustrated the differences in the abundance of characteristic genes among the various groups (**Figure 6I**). To conclude, we classified the cells into groups with high and low scores and utilized GSEA to assess the differences in pathways between these two categories. The high-scoring group exhibited upregulation in pathways such as Lysosome, Complement and Coagulation Cascades, Proteoglycans in Cancer, and Cytokine-Cytokine Receptor Interaction, while downregulation was observed in pathways including Metabolic Pathways and Cell Adhesion Molecules (**Figure 6J**).

**Figure 7:**
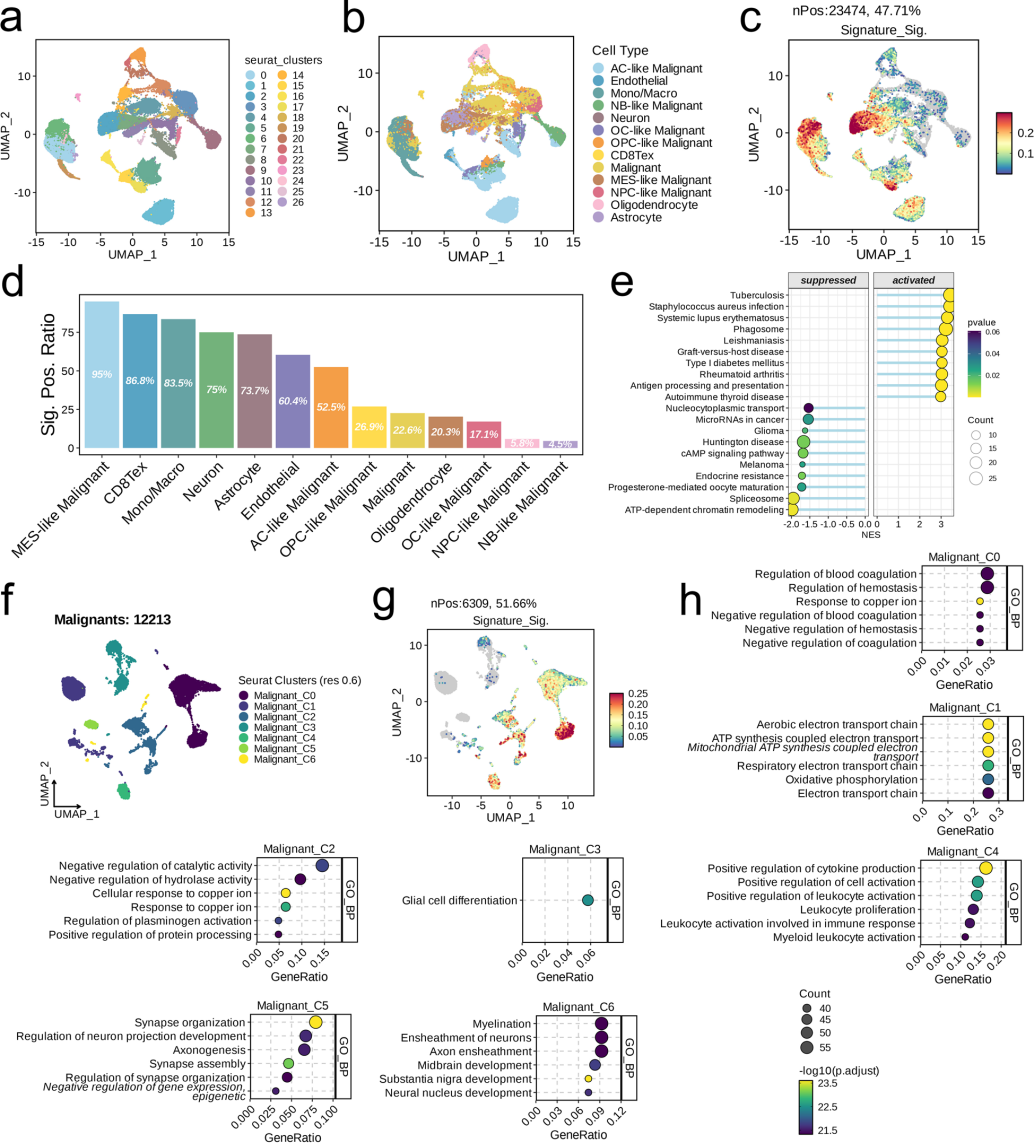
Single-cell analysis of risk score in the integrated LGG/GBM scRNA-seq datasets. **(A–C)** UMAP visualization of single cells from the public LGG/GBM scRNA-seq cohorts. A total of 16 subpopulations were identified under the resolution of 0.6 and manually annotated to nine meta-clusters based on the cranial markers. **(C)** UMAP visualization of the signature scores across cell types. **(D)** The positive ratio of the signature across each cell type. **(E)** GSEA reveals significantly altered pathways in cells with high signature scores compared to those with low scores. **(F)** UMAP showing the subpopulations of malignant cells. **(G)** UMAP visualization of the signature scores across cell types. **(D)** The positive ratio of the signature across each cell type. **(H)** Top six enriched GO terms of each malignant subpopulation.

We integrated three glioma datasets and identified a total of 27 subpopulations at a resolution of 0.6. We categorized these into 13 distinct subpopulations based on their biological characteristics: AC-like Malignant, Endothelial, Mono/Macro, NB-like Malignant, Neuron, OC-like Malignant, OPC-like Malignant, CD8Tex, Malignant, MES-like Malignant, NPC-like Malignant, Oligodendrocyte, and Astrocyte, as illustrated in **Figures 7A, B**. Upon scoring each cell for its signature, we found that high scores were predominantly concentrated in the MES-like Malignant subpopulation (**Figure 7C**). The proportion of signature-positive cells also varied across subpopulations, with the MES-like Malignant subpopulation having the highest (95%) and the NB-like Malignant subpopulation the lowest (4.5%, **Figure 7D**). GSEA results indicated that, compared to cells with low signature scores, those with high signature scores were primarily enriched in pathways such as Phagosome and Antigen Processing and Presentation, while showing downregulation in pathways such as Nucleocytoplasmic Transport, MicroRNAs in Cancer, and Glioma (**Figure 7E**). Further dimensionality reduction clustering of 12,213 cells identified seven subpopulations at a resolution of 0.6 (**Figure 7F**). Signature scoring revealed a concentration of high scores primarily in the Malignant_C0 subpopulation (**Figure 7G**). GO analysis demonstrated the heterogeneity of pathways enriched in each cell subpopulation (**Figure 7H**).

### 3.7 Pan-cancer and immune analysis

**Figure 8:**
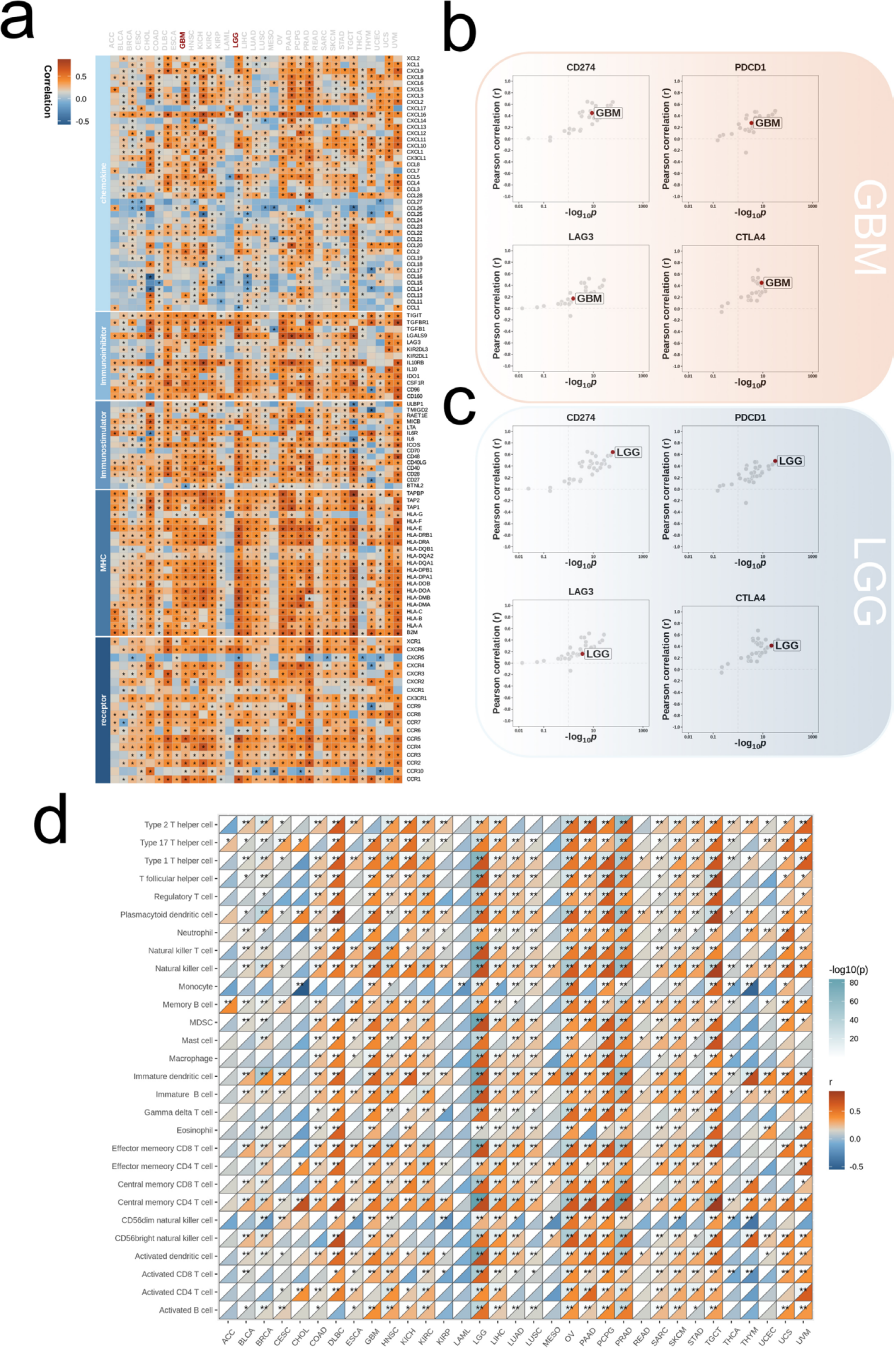
Influence of ALPK1 on immune landscapes in pan-cancer. **(A)** Association of ALPK1 with various immunoregulators (including receptors, MHC molecules, immunostimulators, and chemokines). **(B)** The associations between different tumor types and four immune checkpoints: CD274 (PD-L1), CTLA-4, LAG-3, and PDCD1 (PD-1), with dots representing various cancer types. GBM is marked with a red dot. **(C)** The associations between different tumor types and four immune checkpoints: CD274 (PD-L1), CTLA-4, LAG-3, and PDCD1 (PD-1), with dots representing various cancer types. LGG is marked with a red dot. **(D)** Relationship between ALPK1 and infiltration levels of 28 immune cells in different tumor types, as analyzed by the ssGSEA method. The correlation strength is depicted by color intensity. Statistically significant correlations, determined through Pearson correlation analysis, are marked with asterisks. *p < 0.05; **p < 0.01; ***p < 0.001.

We began our analysis by examining the association between ALPK1 and various immune regulators (including receptors, MHC molecules, immune stimulators, and chemokines) across 33 different tumors (**Figure 8A**). In both glioblastoma multiforme (GBM) and lower-grade glioma (LGG), ALPK1 showed a significant positive association with four immune checkpoints: PD-L1 (CD274), CTLA-4, LAG-3, and PD-1 (PDCD1) (**Figures 8B, C**). Furthermore, ALPK1 showed significant associations with multiple immune cell types across various tumors (**Figure 8D**).

**Figure 9:**
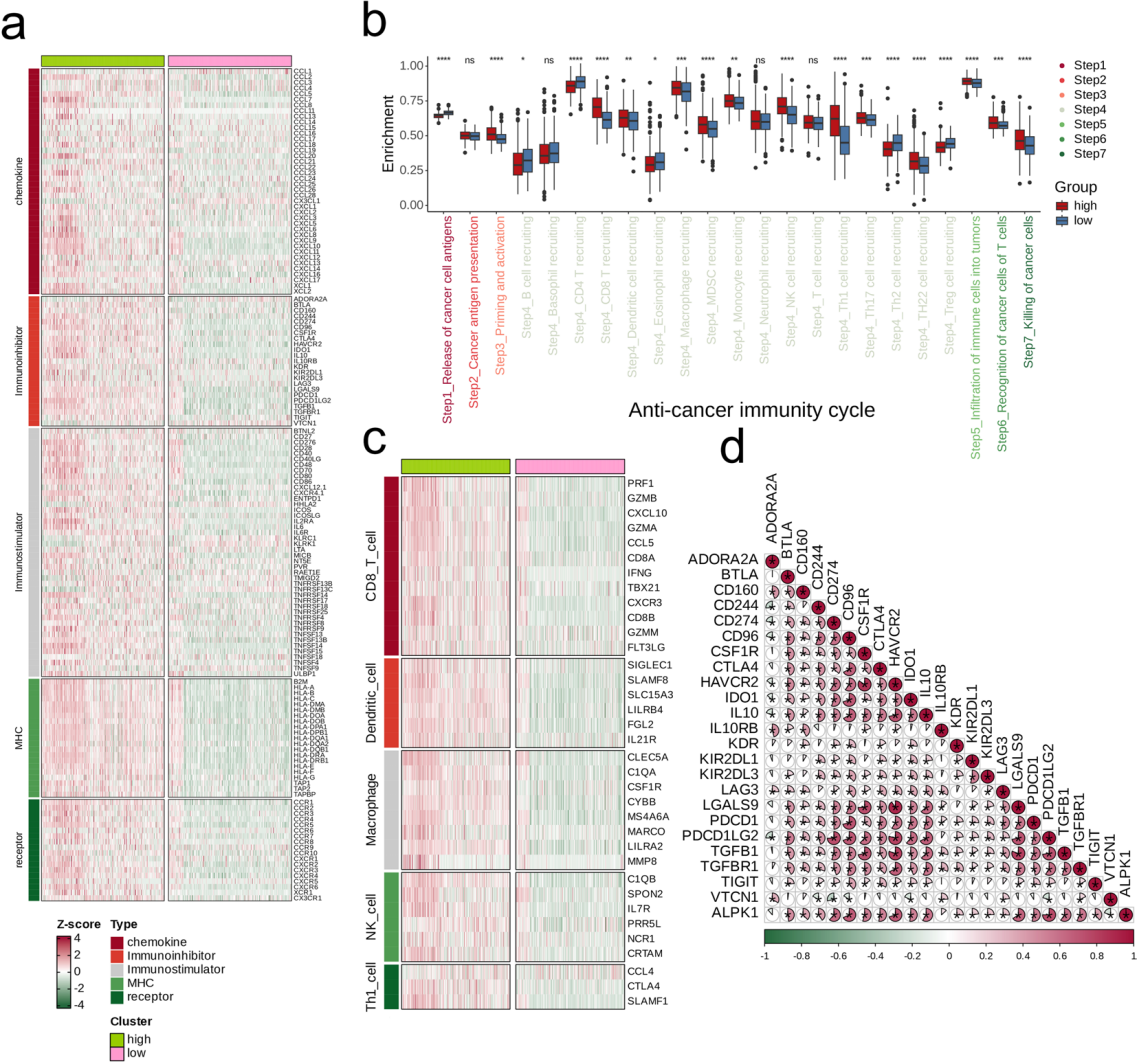
Impact of ALPK1 on the TME in TCGA- LGG/GBM. **(A)** Expression differences of immunoregulators (as identified in **Figure 8A**) between the high- and low- ALPK1 expression groups in TCGA- LGG/GBM. **(B)** Variations in the stages of the cancer immunity cycle for high versus low ALPK1 expression groups. **(C)** Association of ALPK1 with infiltration levels of five types of tumor-infiltrating immune cells: CD8+ T cells, DCs, macrophages, NK cells, and Th1 cells, determined by the six TME decoding algorithms. **(D)** Expression differences in effector genes of these immune cells between the high- and low- ALPK1 groups. Asterisks denote the significance levels as determined by the Mann-Whitney U test. ns, not significant; *p < 0.05; **p < 0.01; ***p < 0.001; ****p < 0.0001.

By stratifying the combined glioma dataset according to the median expression level of ALPK1, we categorized it into groups of high and low expression. We then conducted an initial analysis to examine the differences in immune regulatory factors, presenting the findings in a heatmap (**Figure 9A**). The two expression categories demonstrated variability in enrichment levels throughout various stages of the anti-cancer immunity cycle (**Figure 9B**). The group with high expression consistently showed elevated levels of immune cell-associated effectors in comparison to the group with low expression (**Figure 9C**). Furthermore, a significant positive correlation was observed between ALPK1 and several immune suppressive molecules (**Figure 9D**).

### 3.8 ALPK1 promotes glioma cell proliferation

**Figure 10:**
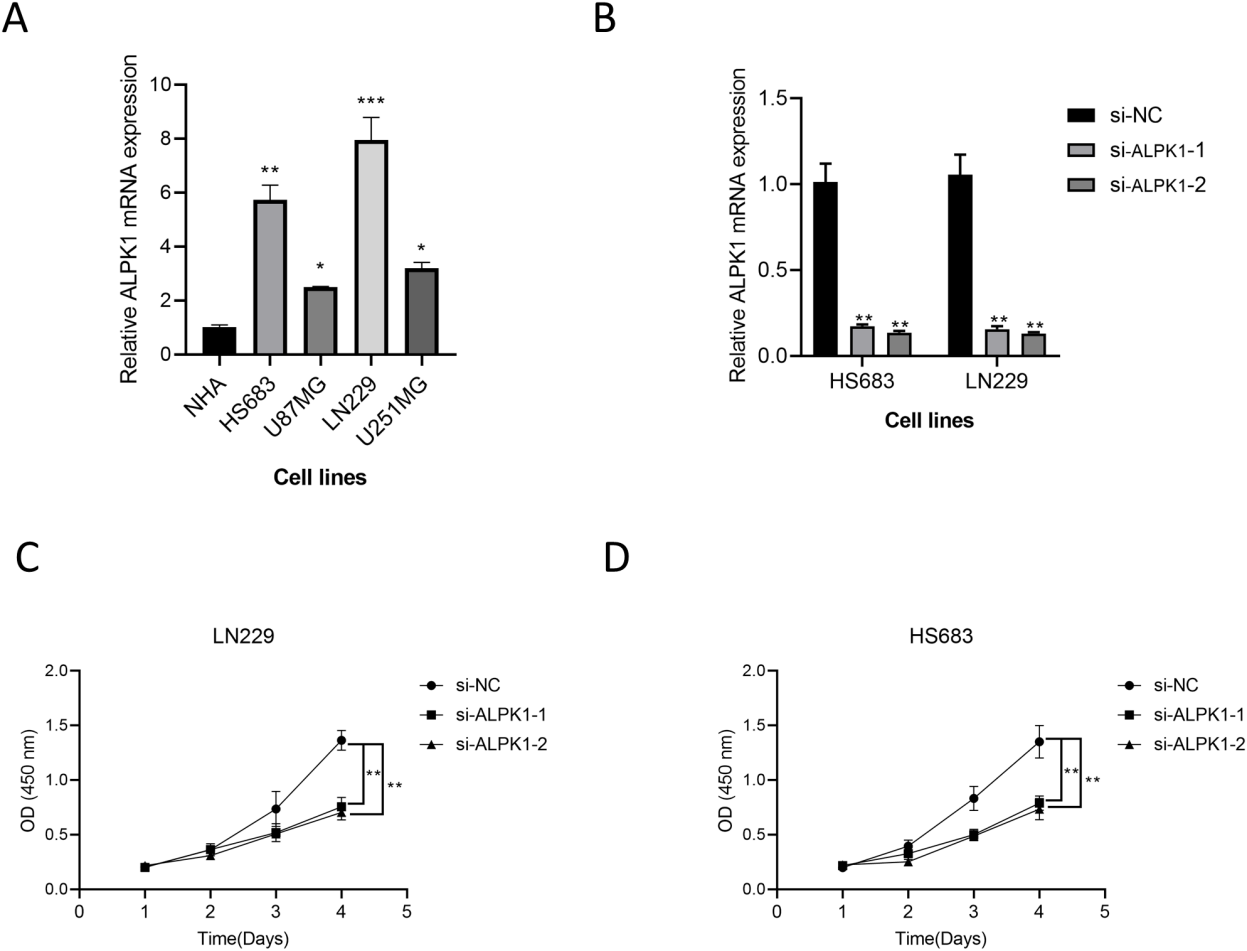
The effect of ALPK1 on Glioma was verified by wet experiment. **(A)** Comparison of mRNA expression levels of ALPK1 among cell lines. **(B)** ALPK1 knock inefficiency assessment. **(C)** Changes in proliferation levels after ALPK1 knockdown in LN229 cell lines. **(D)** Changes in proliferation levels after ALPK1 knockdown in HS683 cell lines. *p < 0.05; **p < 0.01; ***p < 0.001.

There is currently a lack of studies investigating the role of ALPK1 in glioma cells; thus, we selected this gene from our model for experimental validation. Comparative analysis of expression levels among different cell lines revealed that ALPK1 is significantly overexpressed in glioma cell lines (p < 0.05, **Figure 10A**). Subsequently, we achieved effective knockdown of ALPK1 in two cell lines, demonstrating a substantial reduction in expression (p < 0.01, **Figure 10B**). The findings from the CCK8 assay demonstrated that the reduction of ALPK1 notably decreased the growth of tumor cells (p < 0.01, **Figures 10C, D**). Therefore, our findings suggest that ALPK1 plays a critical role in promoting glioma cell proliferation.

## 4 Discussion

Glioma, which is a type of aggressive tumor that arises from glial cells, ranks among the most frequently occurring primary intracranial tumors. It is distinguished by elevated rates of incidence, recurrence, and mortality, along with low rates of successful treatment (23, 24). Its clinical presentation is diverse, and treatment primarily involves surgical resection, supplemented by radiotherapy and chemotherapy (25, 26). Although there have been substantial improvements in the diagnosis and management of gliomas, the outlook for patients continues to be unfavorable, especially for high-grade gliomas, which are characterized by notably brief median survival durations (27, 28). Consequently, exploring molecular subtyping, identifying novel prognostic biomarkers, and understanding the tumor’s immune microenvironment is vital for improving treatment strategies and outcomes.

Nucleotide metabolism is a critical process for cell survival and proliferation, playing an essential role in tumorigenesis. Abnormalities in nucleotide metabolism can facilitate rapid tumor cell proliferation and potentially influence the sensitivity of these cells to treatment (8). Research has indicated a close correlation between alterations in nucleotide metabolism and the development of gliomas, particularly in aspects such as cell cycle regulation, DNA repair, and energy metabolism (29). The precise influence of nucleotide metabolism on the prognosis of glioma, along with its potential as a target for therapy, requires additional research.

In this study, we utilized NMF analysis on gene expression data from glioma patients, categorizing them into two distinct clusters. Notably, patients in cluster 2 exhibited significantly lower survival rates and were, on average, older than those in cluster 1. This suggests that age may be a crucial factor influencing glioma patient survival, with differing biological behaviors and treatment responses among age groups. Moreover, cluster 2 demonstrated a significantly higher expression of glycolytic, amino acid metabolism, and lipid metabolism-related genes compared to cluster 1, indicating that metabolic characteristics may play a key role in distinguishing patients with different prognoses.

To further explore potential biomarkers, we employed WGCNA for initial gene screening (30). By setting a suitable soft threshold, we effectively built a scale-free network and recognized six modules that have substantial associations with clinical pathological characteristics. Notably, the MEcyan module exhibited strong positive correlations with both cluster and mortality, suggesting its close association with disease progression. Functional enrichment analysis of the MEcyan module revealed associations with various biological processes, including immune cell activation, cell junction assembly, synaptic transmission regulation, oligodendrocyte development, and B cell-mediated immune responses.

We created 101 prognostic models utilizing machine learning techniques and, using the average C-index ranking derived from validation cohorts, identified the combination of LASSO and GBM algorithms as the most effective model (31). The model demonstrated significant predictive accuracy and effectiveness across both training and validation datasets. Furthermore, in three distinct datasets, the survival rates observed in the high-risk cohort were substantially lower than those seen in the low-risk cohort, highlighting the reliability of our risk scoring system as a prognostic tool. Additionally, a meta-analysis revealed notable heterogeneity among the three datasets; however, within the high-risk cohorts, risk levels were significantly elevated compared to those in the low-risk groups, thus reinforcing the model’s robustness and applicability. The risk scoring system uncovered distinct gene expression differences between patients classified as high-risk and those deemed low-risk. Specifically, the low-risk individuals showed significantly heightened activity in pathways associated with Androgen, TNFα, JAK-STAT, and VEGF when compared to the high-risk group, highlighting the substantial variability in pathway activity between the two risk categories. These observations imply that the activation of certain signaling pathways might impact patient prognosis and could serve as potential novel targets for therapeutic interventions. Additionally, the risk score correlated significantly with a range of apoptosis-related genes, indicating a potential dysregulation in tumor cell proliferation control mechanisms.

This research also explored the variations in levels of immune cell infiltration among different clusters and risk categories. Our findings revealed that immune cell infiltration levels were significantly higher in cluster 2 and the high-risk category compared to cluster 1 and the low-risk category. Additionally, both the immune and stromal scores were markedly elevated in the high-risk group, indicating a richer presence of immune and stromal components within their tumor microenvironment. Notably, despite the elevated immune cell infiltration and stromal elements observed in cluster 2 and the high-risk group, the prognostic outcomes were contrary. This intensified immune response did not lead to improved results; on the contrary, it correlated with worse clinical outcomes, highlighting the necessity for further investigation into the specific roles and interactions of these immune cells and stromal elements. There could be mechanisms of immune suppression or adverse effects from certain immune cell subpopulations, leading to an active immune response that is ineffective in curbing tumor advancement. This observation offers essential insights for the formulation of future immunotherapeutic approaches.

Patients exhibiting different levels of PD-L1 or CTLA-4 showed that individuals in the high-risk category had a greater likelihood of responding positively to immunotherapy. This indicates that our risk assessment system might also help in pinpointing patients who could gain benefits from immunotherapy. Although direct single-cell datasets on glioma immunotherapy with checkpoint inhibitors are lacking, we inferred the distribution and functional status of immune cells by analyzing lung cancer-related single-cell datasets. By employing the Seurat algorithm for dimensionality reduction clustering, we identified several cell subpopulations, revealing significant differences in signature scores across these groups. Notably, high signature scores were concentrated in myeloid cell subpopulations, with the highest positivity rate in stromal cell subpopulations (89.3%). Additionally, cells with high signature scores showed upregulation in pathways such as lysosome and complement and coagulation cascades, while downregulation occurred in metabolic pathways and cell adhesion molecules. This indicates functional disparities among different cellular states and their roles in disease progression. The activation of lysosomal and complement systems, along with changes in proteoglycans and cytokine networks, may be related to immune evasion mechanisms. Conversely, the downregulation of metabolic pathways and cell adhesion molecules suggests that these cells may have adopted alternative metabolic strategies and potentially lost functions dependent on cell adhesion. This implies a transformation enabling better survival and proliferation within the tumor microenvironment.

Lastly, we conducted a pan-cancer analysis, revealing significant correlations between ALPK1 and various immune checkpoints, including PD-L1, CTLA-4, LAG-3, and PDCD1. This result indicates that ALPK1 could significantly influence the modulation of the immune microenvironment. Studies have shown that ALPK1 can affect the expression of immune-related genes, thereby changing the tumor microenvironment and affecting the development of glioma (32). Additionally, the levels of ALPK1 expression were associated with the infiltration of immune cells in various cancer types, where groups with high expression typically displayed increased concentrations of effector molecules related to immune cells in comparison to those with low expression. Furthermore, research experiments suggest that ALPK1 is vital for enhancing the proliferation of glioma cells. ALPK1 also displayed significant positive correlations with various immune suppressive molecules, potentially indicating its pivotal role in regulating specific stages of the anti-tumor immune cycle.

While this research highlighted variances in the metabolic traits and immune profiles of glioma patients using various data sets and techniques, and established a dependable prognostic risk scoring system, it also presents several limitations. These include inadequate representation of the data sets, restricted generalizability of the machine learning models, and the need for validation of the clinical potential of the ALPK1 gene as a biomarker. Upcoming investigations should concentrate on multi-center clinical trials, analyses of immune cell functions at the single-cell level, and the specific functional mechanisms of the ALPK1 gene to enhance both the applicability of the findings and their clinical significance.

In conclusion, this study utilizes an integrative bioinformatics approach to unveil distinct metabolic features and immune states in glioma patients, establishing a reliable prognostic risk scoring system. Our findings provide new perspectives for further understanding the complex biological mechanisms of gliomas and lay the groundwork for future precision medicine strategies.

## 5 Conclusion

This study integrates various advanced bioinformatics approaches to reveal the heterogeneity of metabolic and immune states among glioma patients. Furthermore, we successfully developed an effective prognostic risk assessment model. These findings not only provide new insights into the complex biological underpinnings of glioma but also lay a crucial foundation for advancing the future of precision medicine.

## Data Availability

The original contributions presented in the study are included in the article/supplementary material. Further inquiries can be directed to the corresponding author.
